# Association Between Pulmonary Vascular Volume and Cardiac Structure and Function in Patients With Atrial Fibrillation

**DOI:** 10.1016/j.amjcard.2023.07.119

**Published:** 2023-08-19

**Authors:** Anne Bjerg Nielsen, Kristoffer Grundtvig Skaarup, Kasper Djernæs, Lisa Steen Duus, Caroline Espersen, Samuel Kiil Sørensen, Martin Huth Ruwald, Morten Lock Hansen, René Husted Worck, Arne Johannessen, Jim Hansen, Pietro Nardelli, Rubén San José Estépar, Raúl San José Estépar, Tor Biering-Sørensen

**Affiliations:** a Department of Cardiology, Herlev & Gentofte Hospital, University of Copenhagen, Denmark;; b Applied Chest Imaging Laboratory, Department of Radiology, Brigham and Women’s Hospital, Harvard Medical School, Boston, Massachusetts;; c Department of Biomedical Sciences, Faculty of Health and Medical Sciences, University of Copenhagen, Copenhagen, Denmark.

**Keywords:** atrial fibrillation, transthoracic echocardiography, computed tomography, pulmonary vascular volume

## Abstract

Pulmonary vascular abnormalities, quantified from computed tomography scans, have frequently been observed in patients with pulmonary diseases. However, little is known about pulmonary vascular changes in patients with cardiac disease. Thus, we aimed to examine the cardiopulmonary relation in patients with atrial fibrillation (AF) by comparing pulmonary vascular volume (PVV) to echocardiographic measures and AF severity. A total of 742 patients (median age 63 years, 70% men) who underwent ablation for AF were included. Preprocedural cardiac computed tomography was used to measure the total and small-vessel PVV, along with the pulmonary artery to aorta ratio and the degree of emphysema. The association between PVV and echocardiographic parameters was evaluated using a multivariable linear regression analysis. Lower total and small-vessel PVV were associated with more impaired measures of cardiac structure and function, including but not limited to left ventricular ejection fraction and peak atrial longitudinal strain. Patients with reduced total and small-vessel PVV had higher odds of having persistent AF than paroxysmal AF in the unadjusted logistic regression analyses. However, after clinical and echocardiographic adjustments, only reduced small-vessel PVV remained independently associated with persistent AF (odds ratio 1.90, 95% confidence interval 1.26 to 2.87, p = 0.002). In conclusion, pulmonary vascular remodeling is associated with persistent AF and with more impaired measures of cardiac structure and function, providing further insights into heart-lung interactions in this patient group.

Atrial fibrillation (AF) is the most common cardiac arrhythmia and is associated with substantial morbidity and mortality.^1^ Patients with AF frequently have coexisting lung disease, including chronic obstructive pulmonary disease (COPD), which can lead to disease progression and increased cardiovascular mortality.^2^ Thus, the close interrelation between the heart and the lungs in patients with AF is worth further exploring. Novel algorithms, based on artificial intelligence, enable automatic segmentation and quantification of pulmonary vascular volume (PVV) and remodeling in computed tomography (CT).^3–5^ Using these techniques, pulmonary vascular alterations, such as the loss of small peripheral pulmonary vasculature, termed vascular pruning, have been recognized as significant pathophysiologic characteristics in various pulmonary diseases.^3,6,7^ Similarly, atrial remodeling, characterized by structural, mechanical, and electrical changes in the atria, is a pathophysiologic characteristic of prolonged AF.^8^ However, the relation between AF and changes in pulmonary vascular morphology remains unknown and may entail important pathophysiologic information regarding AF natural history. Therefore, this study aimed to investigate the cardiopulmonary relation in patients with AF.

## Methods

The study population consisted of 1,823 patients with AF who underwent ablation at Gentofte University Hospital from 2011 to 2018. The clinical characteristics and medical history were obtained from the Danish National Ablation Registry. The inclusion criteria were patients with AF with available preprocedural cardiac CT and transthoracic echocardiography (TTE). The exclusion criteria were no available CT (n = 661), no available TTE (n = 358), inadequate quality of CT for analysis (n = 21), and inadequate image quality of TTE (n = 41), leaving a total of 742 patients for inclusion in the final study population.

The study was performed in compliance with the second Declaration of Helsinki and was approved by the Danish Data Protection Agency and the Danish Patient Safety Authority. Approval from regional ethics committees is not needed for retrospective studies of this sort.

All patients had comprehensive TTE analysis performed using Vivid 9 ultrasound system (GE Healthcare, Horten, Norway). Echocardiograms were stored in a GE Healthcare image vault and analyzed offline by a single investigator with commercially available post-processing software, EchoPAC v202. During TTE, 547 patients (74%) were in sinus rhythm, whereas 195 (26%) had ongoing AF. The detailed method of echocardiographic measurements is described in the Supplementary Material.

All patients underwent ECG-gated contrast-enhanced cardiac CT. A total of 3 different scanners were used: Aquilion ONE (Toshiba, Tokyo, Japan) (n = 344 [46%]), Brilliance 64 (Philips, Cleveland, Ohio) (n = 317 [43%]), and SOMATOM Definition Flash (Siemens, Munich, Germany) (n = 81 [11%]). For Toshiba, images were reconstructed using an FC04, FC08, or FC18 algorithm at 0.5- or 1.0-mm slice thickness. Philips CT images were reconstructed using a C algorithm at 2.0-mm slice thickness. Images from Siemens were reconstructed using I26f, I30f, or I36f at 2.0- or 3.0-mm slice thickness.

The CT image processing was performed as previously outlined.^3,9^ In brief, the automatic lung segmentation was performed using the Chest Imaging Platform software (http://www.chestimagingplatform.org). This system uses deep learning methods to detect, classify, and measure vessels on CT scans. Using these techniques, pulmonary vessels were automatically identified and segmented from cardiac CT to create 3-dimensional reconstructions of the intraparenchymal pulmonary vasculature, including the entire vessel (wall and lumen) of both arterial and venous blood vessels. The radius of each artery and vein was measured using a sizing deep learning approach that provides accurate measurement at the subvoxel resolution.^5^ Manual quality assurance was performed to verify the intermediate results of the processing pipeline, including lung mask verification, exclusion of scans with artifacts, and visual verification of the total vascular reconstruction. The morphologic assessment of the pulmonary vasculature included total blood volume (TBV) and the aggregate blood volume for vessels <10 mm^2^ in cross-sectional area (BV10). To account for anatomic variability in PVVs, the measurements were normalized to the total lung volume (TLV) to calculate the total pulmonary vascular volume (TBV/TLV) and small vessel fraction (BV10/TLV). The BV10/TLV represented a measure of the relative distribution of blood volume in the peripheral pulmonary vessels, reflecting a radiographic measure of pulmonary vascular pruning, with lower values indicating more extensive vascular pruning. To evaluate the percentage of emphysematous lung tissue, low attenuation densitometric measures were obtained at the density threshold of −950 Hounsfield units (%LAA–950). The pulmonary artery and ascending aortic diameters and the pulmonary artery to aortic diameter ratio (PA:A) were manually measured using the Chest Imaging Platform for 3D Slicer^10^ at the level of the pulmonary artery bifurcation.

STATA version 16.1 (StataCorp, College Station, Texas) was used for all statistical analyses. Clinical and echocardiographic characteristics of the study population were stratified by quartiles of TBV/TLV and BV10/TLV. Continuous data exhibiting Gaussian distribution were compared with analysis of variance and presented as means ± SD. Non-Gaussian distributed continuous variables were compared using the Kruskal–Wallis test and reported as median and interquartile range (IQR). Pearson’s chi-square test was used to compare proportions. Q-Q plots were visually inspected to test for Gaussian distribution. A multivariable linear regression analysis adjusted for age, gender, heart rate, AF during TTE, and emphysema was used to examine the relation between pulmonary vascular morphology and clinical and echocardiographic characteristics, the %LAA–950, and PA:A. Restricted cubic spline curves were constructed to visualize the association between the total and small-vessel PVV and selected echocardiographic measures with the number of knots determined by the lowest Akaike information criterion.

To explore the association between AF severity and total and small-vessel PVV, a univariable linear regression was used to test for trends and the Wilcoxon rank-sum test was used for pairwise comparisons of median TBV/TLV and BV10/TLV between AF subtypes (paroxysmal AF (PAF) and persistent AF). In addition, a logistic regression analysis was used to test the association between AF subtype and TBV/TLV and BV10/TLV dichotomized at the median value of the population. Effect estimates were expressed as odds of having persistent AF for patients with the most versus the least loss of blood volume in the pulmonary vessels (patients in the lowest vs the highest 50% of TBV/TLV and BV10/TLV). Fitted odds ratios were obtained with multivariable logistic regression analysis by adjusting for age, gender, heart rate, AF during TTE, emphysema, CHA_2_DS_2_-VASc score (score of congestive heart failure, hypertension, age, diabetes, stroke, transient ischemic attack, thromboembolism, vascular disease, and sex category), left atrial (LA) diameter, and left ventricular (LV) ejection fraction (LVEF). Logistic restricted cubic spline curves were created to visualize the association between AF severity and total and small-vessel PVV using the lowest Akaike information criterion to determine the number of knots. p <0.05 were considered statistically significant in all analyses.

## Results

Of the 742 patients included, 523 were men (70%), median age was 63 years (IQR 56 to 70, range 29 to 85), and the median body mass index was 26 kg/m^2^ (IQR 24 to 29). The median burden of emphysematous lung tissue was 0.06% (IQR 0.02 to 0.19). A total of 422 patients (60%) had PAF, whereas 300 had persistent AF (40%), with 78 being long-standing persistent AF. Co-morbidities in the study population included hypertension (40%); ischemic heart disease (7%); diabetes (6%); congestive heart failure (10%); and a history of stroke, transient ischemic attack, or thromboembolism (7%).

The association between BV10/TLV quartiles and baseline characteristics is listed in Table 1, along with standardized *β* coefficients adjusted for age, gender, heart rate, AF during TTE, and emphysema. Higher BV10/TLV was associated with lower age, gender (less often men), lower body mass index, and AF subtype (more often PAF) in the adjusted analyses. Additionally, after adjustments, patients with a higher BV10/TLV had a significantly lower CHA_2_DS_2_-VASc score, less often subjective dyspnea, and a lower prevalence of congestive heart failure. In the adjusted analyses, BV10/TLV demonstrated associations with echocardiographic measures, including the maximal tricuspid regurgitation velocity, LVEF, LV end-systolic and end-diastolic volumes, the ratio of early transmitral inflow velocity to early diastolic strain rate, and LA structural and functional measurements.

**Figure 1.**
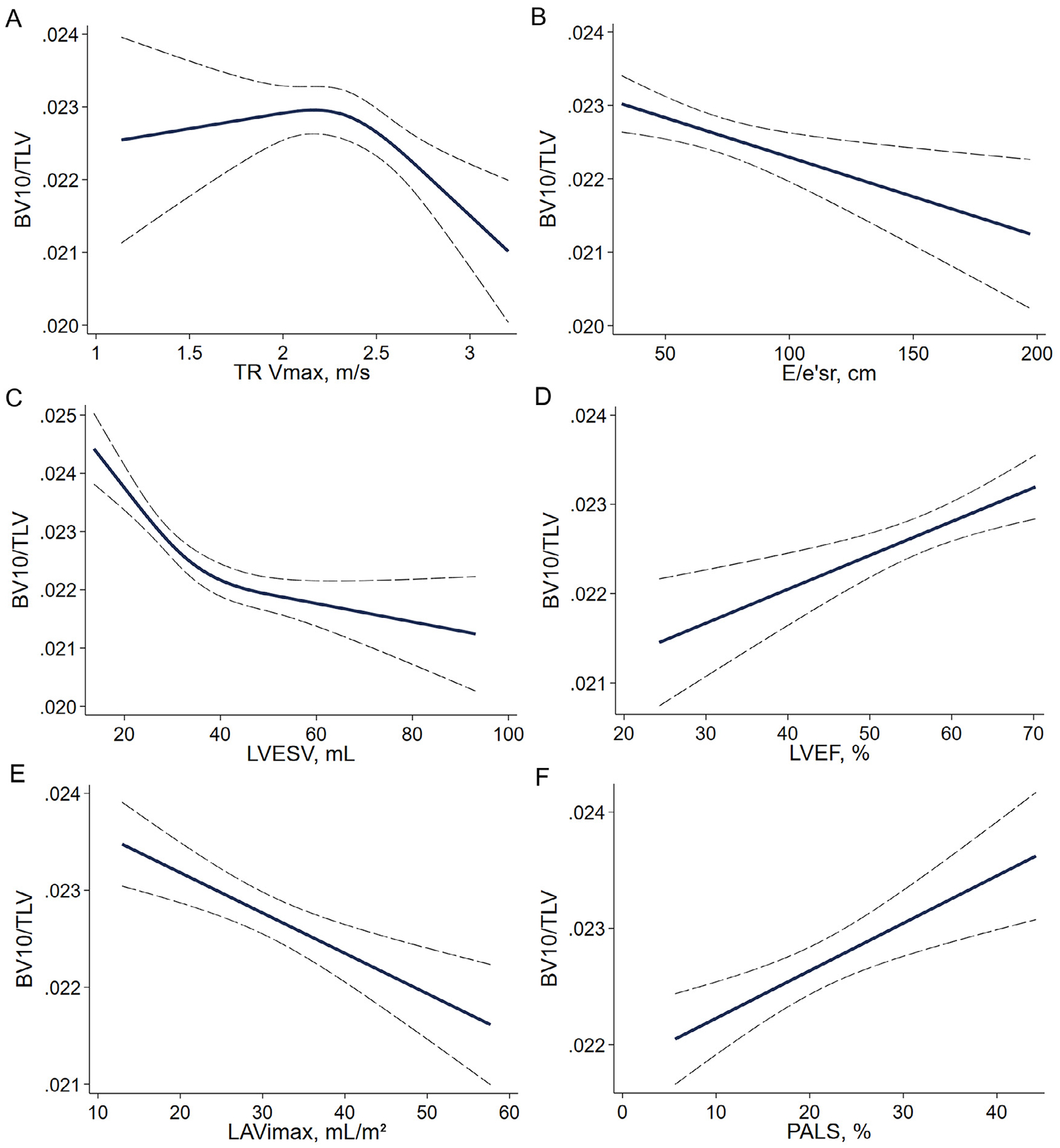
Association between small vessel fraction and cardiac structure and function. Restricted cubic splines displaying the unadjusted associations between BV10/TLV and (*A*) TR *V*_max._, (*B*) E/e’sr, (*C*) LVESV, (*D*) LVEF, (*E*) LAVi_max_, and (*F*) PALS with 95% confidence intervals. E/e′ sr = ratio of early mitral inflow velocity to early diastolic strain rate; LAVi_max_ = maximum left atrial volume index; LVESV = left ventricular end-systolic volume; PALS = peak atrial longitudinal strain; TR Vmax = maximal tricuspid regurgitation velocity.

Patients with a higher BV10/TLV also had a lower PA:A and lower degrees of emphysema after adjustments. Figure 1 illustrates the association between BV10/TLV and selected echocardiographic measures of the right ventricle (RV), LV, and LA.

**Figure 2.**
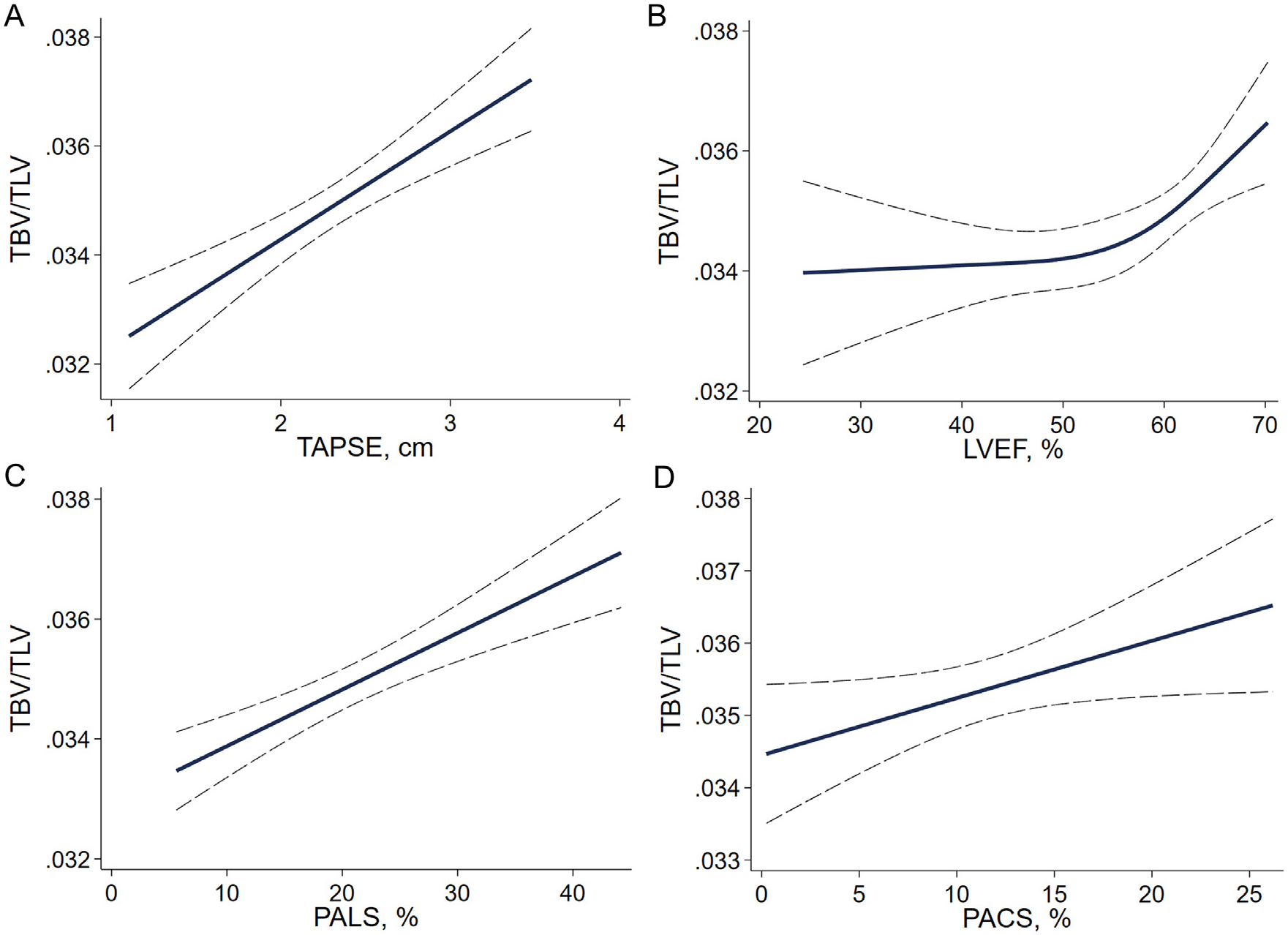
Association between total pulmonary vascular volume and cardiac function. Restricted cubic splines displaying the unadjusted associations between TBV/TLV and (*A*) TAPSE, (*B*) LVEF, (*C*) PALS, and (*D*) PACS with 95% confidence intervals. PACS = peak atrial contraction strain; PALS = peak atrial longitudinal strain; TAPSE = tricuspid annulus plane systolic excursion.

Supplementary Table 1 presents the association between TBV/TLV quartiles and clinical and echocardiographic parameters, along with results from multivariable linear regressions. After adjustments, patients with a higher TBV/TLV were younger, more often men, and more often had PAF. Higher TBV/TLV was also associated with a lower prevalence of stroke, transient ischemic attack, or thromboembolism and less subjective dyspnea. In the adjusted analyses, higher TBV/TLV was associated with increased RV and LV systolic function, measured by the tricuspid annular plane systolic excursion and LVEF, respectively. LA function, assessed by peak atrial longitudinal strain and peak atrial contraction strain, also increased with higher TBV/TLV, whereas the degree of emphysematous lung tissue was lower. Figure 2 presents the association between TBV/TLV and selected echocardiographic measurements.

**Figure 3.**
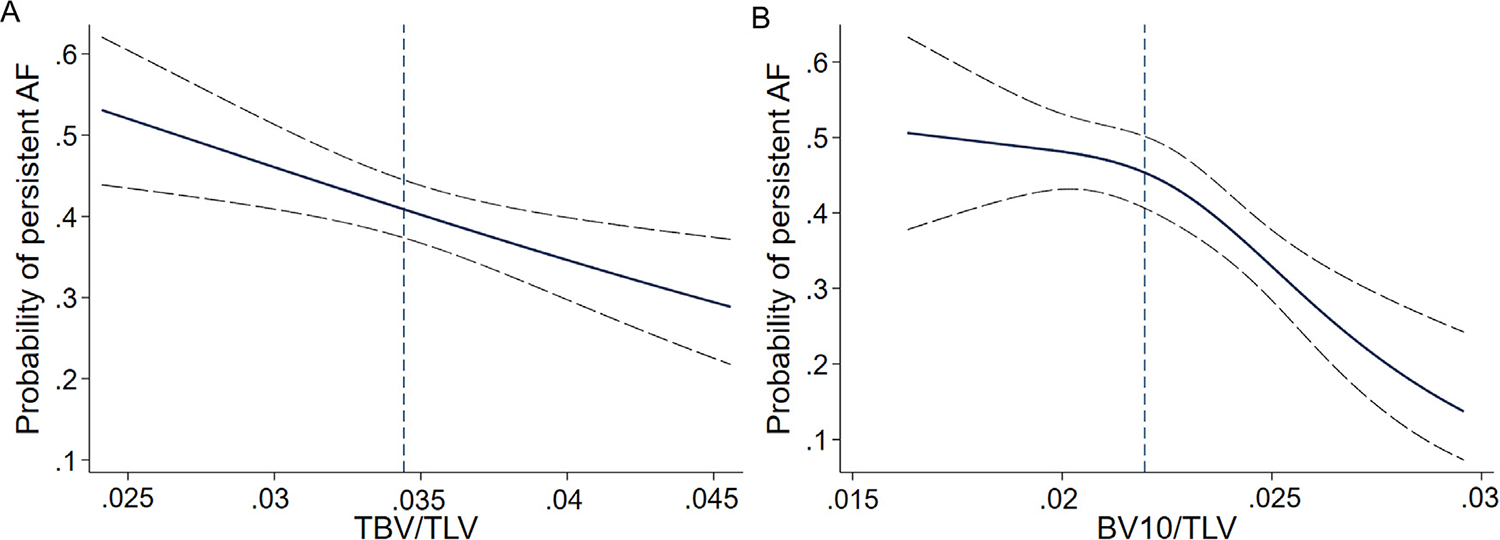
Relation between pulmonary vascular volume and AF subtype. Logistic restricted cubic spline curves displaying (*A*) TBV/TLV and (*B*) BV10/TLV in relation to the probability of having persistent AF. The vertical dashed lines represent the optimal cut-points in AF severity classification.

Patients with persistent AF had lower TBV/TLV and BV10/TLV in trend and pairwise analyses than patients with PAF (Supplementary Figure 1). In the univariable logistic regression analyses, both reduced TBV/TLV and BV10/TLV were associated with increased odds of having persistent AF. However, after the adjustments, only lower BV10/TLV, indicating more extensive vascular pruning, remained independently associated with 1.9-fold higher odds of having persistent AF than PAF (odds ratio 1.90, 95% confidence interval 1.26 to 2.87, p = 0.002) (Table 2). Figure 3 displays the distribution of TBV/TLV and BV10/TLV in relation to the probability of having persistent AF compared to PAF.

## Discussion

To the best of our knowledge, this study is the first to use state-of-the-art CT imaging techniques combined with echocardiography to examine the relation between pulmonary vascular characteristics and cardiac structure and function in patients with AF. We established that in patients with AF, reduced blood volume in the peripheral pulmonary vasculature, indicating more extensive pruning, was associated with increased RV systolic pressure, reduced LV systolic function, reduced LA function, and increased LV and LA volumes and LV filling pressure. We also established that reduced total pulmonary blood volume was associated with reduced RV and LV systolic function and reduced LA function. Moreover, we demonstrated that patients with reduced peripheral pulmonary blood volume (more extensive vascular pruning) had higher odds of having persistent AF than PAF. This study contributes to a further understanding of the pathophysiology of AF and the heart-lung interactions in this common arrhythmic disorder. Also, it adds to the growing body of literature describing the complex cardiopulmonary system in general and in patients with AF. Finally, our study exemplifies a new frontier of research that would have been infeasible or too time-consuming to perform before the maturation of automatic approaches.

As stated, we observed an association between persistent AF and more extensive pruning of the peripheral pulmonary vasculature, even after adjusting for clinical and echocardiographic characteristics that have shown to differ between AF subtypes.^11^ In a recent systematic review and meta-analysis, COPD was recognized as an independent risk factor for AF progression and recurrence.^12^ This is consistent with our findings as it is well-established that COPD is characterized by pruning of the small pulmonary vessels.^3^ Thus, a link between peripheral vascular alterations and AF severity appears to exist, regardless of the low degree of emphysematous changes in this AF population. However, it remains unclear whether vascular pruning is a contributing factor to the progression of AF or a consequence of more advanced disease and greater disease burden.

Previous studies have reported varying results on the correlation between PVV and emphysema. Similar to the observations of this study, emphysema estimated by %LAA–950 correlated with pruning in participants with smoking-related COPD in the COPDGene study.^3^ However, Synn et al^13^ found no correlation between vascular pruning and visual emphysema in the Framingham Heart Study but observed associations between PVVs and spirometry-measured lung health in the generally healthy adults without high burdens of smoking or lung disease. The discrepancies between these findings may arise from the differences in emphysema assessment between quantitative approaches (%LAA–950) and qualitative approaches such as visual assessment. Our study indicates that even in populations with a low emphysema burden, both TBV/TLV and BV10/TLV are inversely related to CT-measured low attenuation areas (%LAA–950). This is consistent with the unsurprising finding that self-reported dyspnea was less frequent with increasing TBV/TLV and BV10/TLV.

We observed inverse associations between BV10/TLV and both the maximal tricuspid regurgitation velocity (an estimate of the pulmonary artery systolic pressure^14^) and PA:A, suggesting that reduced blood volume in the small pulmonary vessels could be related to enlargement and increased pressure of the pulmonary artery. As in COPD, this could be due to a redistribution of the blood flow to larger pulmonary vessels.^15^ Furthermore, the small vessel fraction was associated with LVEF and inversely with LV volumes, suggesting an influence of vascular pruning on both LV function and volumes. TBV/TLV showed associations with LVEF and tricuspid annular plane systolic excursion, demonstrating the relation between pulmonary vascular remodeling and systolic function of both the LV and RV. Notably, no association was observed between the global longitudinal strain and either of the pulmonary vascular metrics. In a recent study, Wells et al^16^ examined the association between PVV and ventricular metrics by cardiac magnetic resonance imaging in a cohort of 24 patients with COPD. Although they found that a lower small vessel fraction correlated with increased RV mass and volumes, they did not observe significant associations with LV structure or function, possibly owing to the small sample size.^16^

Echocardiographic measures of the LA were also associated with pulmonary vascular alterations. Reduced TBV/TLV was linked to lower LA reservoir and contractile function, whereas BV10/TLV, after multivariable adjustments, was associated with all LA structural and functional parameters, suggesting a clear relation between vascular pruning and the LA in these patients. In addition, reduced BV10/TLV was associated with a higher ratio of early transmitral inflow velocity to early diastolic strain rate, a sensitive indicator of increased LV filling pressure,^17^ consistent with the association found between BV10/TLV and LV and LA volumes.

The pathophysiologic mechanisms that link pulmonary alterations to cardiac structure and function in patients with AF are yet to be determined. Hemodynamic abnormalities are likely to play a role in the associations because of the close anatomic connection. Inflammatory mediators and oxidative stress are associated with both AF and COPD and may be a contributing factor to the observed relation.^18^ However, it remains uncertain to what extent pulmonary vascular changes in this patient population are because of the changes in preload, vasoconstriction, destruction of pulmonary vessels, inflammatory processes related to endothelial dysfunction,^19^ or combinations of multiple factors. Additional studies are needed to further investigate this complex interaction between cardiac structure and function and pulmonary vascular remodeling in patients with AF.

One limitation of this study is that PVVs and emphysema were measured on the lung fields of cardiac CT scans, limiting the assessment in the apical lung sections. However, previous studies have found that assessing emphysema in cardiac CT scans is comparable to full lung scans and that the greatest blood flow occurs in the central and lower parts of the lungs independent of gravity.^20,21^ Also, this degree of exclusion was equal for all study participants, making it comparable across the study population. Another limitation is the cross-sectional method and the associated risk of reverse causality. Therefore, we cannot make conclusions about the directionality between cardiac and pulmonary remodeling in this patient population and additional studies are, therefore, needed. Finally, we did not have information available regarding chronic pulmonary diseases, smoking, or direct measures of pulmonary function, such as the forced expiratory volume in 1 second and forced vital capacity, for this study population to include in our models. However, the degree of emphysema, which has proved to significantly correlate with lung function in previous studies,^22^ was measured on all CT scans and included in the models.

In conclusion, reduced blood volume in the total and peripheral pulmonary vascular bed is associated with more impaired cardiac structural and functional measures in patients with AF. Moreover, patients with reduced blood volume in the peripheral pulmonary vessels, indicating more extensive vascular pruning, have higher odds of having persistent AF than PAF. This study contributes to a further understanding of the cardiopulmonary relation in patients with AF, which after additional investigation could lead to improved diagnostic tools and personalized treatment strategies.

## Supplementary Material

Supplement

Supplementary material associated with this article can be found in the online version at https://doi.org/10.1016/j.amjcard.2023.07.119.

## Figures and Tables

**Table 1:** Baseline characteristics of patients stratified by BV10/TLV

	BV10/TLV <0.021 (n=186)	BV10/TLV 0.021–0.023 (n=185)	BV10/TLV 0.023–0.024 (n=186)	BV10/TLV >0.024 (n=185)	P-value	Std. Beta*	Adjusted p-value*
**Clinical characteristics**							
Age, years	62.6 (56.6; 69.4)	62.8 (55.6; 69.5)	63.5 (55.1; 69.5)	61.8 (55.1; 69.5)	0.54	−0.109	**0.003**
Male, n (%)	153 (82%)	145 (78%)	130 (70%)	95 (51%)	**<0.001**	−0.287	**<0.001**
BMI, kg/m^2^	27.4 (24.1; 30.8)	27.2 (24.2; 29.6)	26.3 (23.8; 29.0)	25.6 (23.5; 27.7)	**0.002**	−0.240	**<0.001**
AF subtype, n (%)					**<0.001**	−0.141	**<0.001**
Paroxysmal AF	92 (49%)	98 (53%)	117 (63%)	135 (73%)			
Persistent AF	94 (51%)	87 (47%)	69 (37%)	50 (27%)			
**Medical history, n (%)**							
Hypertension	81 (44%)	72 (39%)	81 (44%)	60 (32%)	0.092	−0.056	0.117
Diabetes mellitus	12 (6%)	13 (7%)	12 (6%)	7 (4%)	0.55	−0.024	0.491
Stroke, TIA or thromboembolism	19 (10%)	12 (6%)	11 (6%)	10 (5%)	0.25	−0.060	0.088
Congestive heart failure	25 (13%)	30 (16%)	15 (8%)	7 (4%)	**<0.001**	−0.100	**0.005**
Ischemic heart disease	15 (8%)	14 (8%)	13 (7%)	8 (4%)	0.48	−0.012	0.727
Report of dyspnea	155 (83%)	150 (81%)	143 (77%)	130 (70%)	**0.012**	−0.076	**0.030**
CHA_2_DS_2_-VASc score					0.10	−0.122	**0.008**
0	44 (23%)	54 (29%)	49 (26%)	53 (29%)			
1	57 (31%)	51 (28%)	52 (28%)	47 (25%)			
2	47 (25%)	39 (21%)	47 (25%)	47 (25%)			
3	16 (9%)	30 (16%)	26 (14%)	29 (16%)			
≥ 4	22 (12%)	11 (6%)	12 (7%)	9 (5%)			
**Echocardiography of right ventricle**							
TAPSE, cm (SD)	2.3 (0.6)	2.3 (0.6)	2.3 (0.5)	2.4 (0.4)	0.39	0.013	0.795
TR Vmax, m/s	2.5 (2.2; 2.7)	2.3 (2.1; 2.5)	2.3 (2.1; 2.5)	2.3 (2.1; 2.5)	**0.023**	−0.128	**0.017**
**Echocardiography of left ventricle**							
LVEF, %	57.1 (50.9; 62.7)	58.3 (50.8; 62.7)	58.1 (52.4; 63.7)	61.0 (52.8; 64.4)	**0.012**	0.090	**0.038**
GLS, %	16.3 (12.8; 18.9)	15.8 (13.1; 18.9)	16.7 (12.8; 19.1)	17.0 (14.1; 19.4)	0.13	0.062	0.183
LVEDV, mL	82.5 (70.1; 100.8)	83.9 (67.6; 101.3)	76.2 (62.3; 97.5)	70.0 (55.9; 85.1)	**<0.001**	−0.206	**<0.001**
LVESV, mL	35.0 (26.9; 45.4)	35.5 (28.3; 45.1)	32.4 (25.4; 40.4)	27.9 (22.2; 36.5)	**<0.001**	−0.165	**<0.001**
E/e’sr, cm	66.5 (54.8; 85.9)	64.2 (51.2; 80.7)	64.3 (50.6; 82.1)	59.1 (47.7; 77.5)	**0.026**	−0.105	**0.013**
LV mass index, g/m^2^	91.8 (78.9; 108.4)	90.2 (78.9; 110.1)	91.9 (76.5; 105.6)	84.9 (71.9; 101.4)	**0.022**	−0.039	0.339
**Echocardiography of left atrium**							
LAD, cm	4.1 (3.8; 4.4)	4.0 (3.7; 4.4)	3.9 (3.6; 4.3)	3.8 (3.5; 4.2)	**<0.001**	−0.153	**<0.001**
LAV_max_, mL	67.1 (53.0; 83.0)	62.0 (50.9; 78.2)	58.9 (46.9; 70.4)	55.1 (42.1; 70.5)	**<0.001**	−0.205	**<0.001**
LAV_min_, mL	42.4 (30.5; 60.2)	40.2 (28.7; 55.8)	36.8 (27.5; 47.2)	31.6 (23.3; 47.3)	**<0.001**	−0.177	**<0.001**
LAVi_max_, mL/m^2^	31.1 (24.7; 39.1)	30.6 (24.0; 37.2)	29.0 (23.3; 34.7)	26.9 (21.9; 35.0)	**0.009**	−0.141	**<0.001**
LAVi_min_, mL/m^2^	19.1 (14.2; 26.2)	19.4 (13.4; 28.1)	17.9 (13.5; 23.8)	16.4 (11.8; 23.2)	**0.019**	−0.134	**0.001**
PALS, %	18.7 (12.2; 25.2)	19.5 (12.8; 27.0)	21.1 (12.7; 27.0)	23.0 (15.2; 28.8)	**0.021**	0.141	**0.002**
PACS^†^, %	11.1 (7.6; 14.6)	11.8 (7.9; 15.0)	11.9 (8.8; 14.8)	11.6 (7.2; 15.5)	0.67	0.090	**0.034**
LACS, %	11.3 (9.0; 14.5)	11.8 (9.1; 15.5)	12.1 (9.1; 16.0)	12.8 (9.3; 17.5)	0.089	0.097	**0.013**
**Computed tomography**							
Pulmonary artery diameter, mm	27.6 (25.0; 30.7)	27.8 (25.3; 31.0)	26.1 (24.1; 28.9)	25.4 (22.9; 27.9)	**<0.001**	−0.230	**<0.001**
Ascending aortic diameter, mm	34.7 (32.2; 37.1)	34.4 (31.7; 37.0)	33.1 (30.7; 35.6)	32.3 (29.8; 34.5)	**<0.001**	−0.177	**<0.001**
PA:A	0.8 (0.7; 0.9)	0.8 (0.7; 0.9)	0.8 (0.7; 0.9)	0.8 (0.7; 0.9)	0.49	−0.100	**0.005**
%LAA-950	0.098 (0.017; 0.29)	0.063 (0.024; 0.19)	0.054 (0.015; 0.21)	0.046 (0.019; 0.12)	**0.003**	−0.131	**<0.001**

Data are expressed as median (IQR) unless otherwise specified.

* Adjusted for age, gender, heart rate, AF during echocardiography, and emphysema.

† Only includes patients in sinus rhythm during echocardiography (n=547).

AF = atrial fibrillation; BMI = body mass index; BV10/TLV = blood volume of vessels <10 mm^2^ in cross-sectional area normalized to total lung volume; CHA_2_DS_2_-VASc = score of congestive heart failure, hypertension, age, diabetes, stroke, transient ischemic attack, thromboembolism, vascular disease, and sex category; E/e’sr = ratio of early mitral inflow velocity to early diastolic strain rate; GLS = global longitudinal strain; LACS = left atrial strain during conduit phase; LAD = left atrial diameter; LAV = left atrial volume; LAVi = left atrial volume index; LV = left ventricular; LVEDV = left ventricular end-diastolic volume; LVEF = left ventricular ejection fraction; LVESV = left ventricular end-systolic volume; PACS = peak atrial contraction strain; PALS = peak atrial longitudinal strain; PA:A = ratio of pulmonary artery diameter to ascending aortic diameter; TAPSE = tricuspid annulus plane systolic excursion; TIA = transient ischemic attack; TR Vmax = maximal tricuspid regurgitation velocity; %LAA-950 = percentage of emphysematous lung tissue.

**Table 2:** Association between pulmonary vascular volume and AF subtype

	*Univariable analysis*			*Multivariable analysis*		
	OR	95% CI	*p-value*	OR	95% CI	*p-value*
TBV/TLV (low vs. high)	1.37	1.02–1.84	***0.036***	1.28	0.85–1.95	*0.239*
BV10/TLV (low vs. high)	2.02	1.50–2.72	<***0.001***	1.90	1.26–2.87	***0.002***

The multivariable model is adjusted for age, gender, heart rate, AF during echocardiography, emphysema, CHA_2_DS_2_-VASc score, LA diameter, and LVEF. TBV/TLV and BV10/TLV are dichotomized at the median value. Effect estimates are expressed as odds of having persistent AF in patients with lower total and small-vessel pulmonary vascular volume compared to patients with higher volumes.

AF = atrial fibrillation; BV10/TLV = blood volume of pulmonary vessels <10 mm^2^ in cross-sectional area normalized to total lung volume; CHA_2_DS_2_-VASc = score of congestive heart failure, hypertension, age, diabetes, stroke, transient ischemic attack, thromboembolism, vascular disease, and sex category; CI = confidence interval; LA = left atrial; LVEF = left ventricular ejection fraction; OR = odds ratio; TBV/TLV = total blood volume normalized to total lung volume; %LAA-950 = percentage of emphysematous lung tissue.

## Data Availability

The data underlying this article cannot be shared publicly owing to the privacy of patients that participated in the study. The data will be shared on reasonable request to the corresponding author after legal permission from relevant entities.
