# Supplementary material for: Association Between Pulmonary Vascular Volume and Cardiac Structure and Function in Patients With Atrial Fibrillation
Source: Am J Cardiol. Author manuscript; Available in PMC 2026 Jul 6. (PMC13335315; doi:10.1016/j.amjcard.2023.07.119)

SUPPLEMENTAL MATERIAL

Supplementary Methods

Conventional Echocardiography

In the parasternal long-axis view left ventricular (LV) dimensions and left atrial diameter (LAD) were obtained from an angle perpendicular to the structures. Interventricular septal thickness, LV internal diameter, and posterior wall diameter at end-diastole were measured and used to calculate LV mass. LV mass index was obtained by indexing LV mass to body surface area.

In the apical 4- and 2-chamber views LV end-systolic and end-diastolic volumes (LVESV and LVEDV, respectively) were measured, and LV ejection fraction (LVEF) was calculated using the modified biplane Simpson's method. Left atrial (LA) volumes were measured using the biplane area-length method, excluding the pulmonary veins, LA appendage, and mitral leaflets. LA maximal volume (LAV_{max}) was measured at end-systole and LA minimal volume (LAV_{min}) at end-diastole. LA volumes were indexed to body surface area to obtain the maximal and minimal LA volume index ($LAVi_{max}$ and $LAVi_{min}$, respectively).

Transmitral inflow velocities (E, A, E/A, and deceleration time of E-wave) were measured in the apical 4-chamber view using pulsed-wave Doppler. In the apical 4-chamber view, the tricuspid annular plane systolic excursion (TAPSE) was measured using m-mode through the tricuspid lateral annulus. Continuous-wave Doppler of the tricuspid regurgitation trace was used to measure the maximal tricuspid regurgitation velocity (TR Vmax).

Two-Dimensional Speckle Tracking Echocardiography

We performed speckle tracking echocardiography of the LV in apical 4-chamber, 2-chamber, and long-axis views. In all views, the LV wall was divided into 6 segments resulting in a global 18-segment, although segments were omitted if the investigator deemed them untraceable. A semiautomatic function was utilized to track the endocardial border and define a region of interest, which was adjusted manually in case of inaccurate tracing. Global peak systolic longitudinal strain

(GLS) was calculated as the mean of all segments of the LV. Early diastolic strain rate (e'sr) was indexed to early transmitral inflow velocity (E) to obtain E/e'sr.

Speckle tracking of the LA was performed in the apical 4-chamber and 2-chamber views using LV end-diastole as the reference point. Peak atrial longitudinal strain (PALS) was measured as the peak positive strain and represents the LA reservoir function. In patients in sinus rhythm during TTE, peak atrial contraction strain (PACS) was measured at the ECG p-wave, and left atrial strain during the conduit phase (LACS) was calculated as the difference between PALS and PACS. Since LA contraction only occurs in sinus rhythm, PACS could not be measured in patients with ongoing AF and thus, LACS was equivalent to PALS in these patients. All strain values are reported as absolute values.

Supplementary Table 1: Baseline characteristics of patients stratified by TBV/TLV

	**TBV/TLV < 0.032 (n = 186)**	**TBV/TLV 0.032-0.035 (n = 185)**	**TBV/TLV 0.035-0.038 (n = 186)**	**TBV/TLV > 0.038 (n = 185)**	**P-value**	**Std. Beta***	**Adjusted p-value***
**Clinical characteristics**							
Age, years	67.5 (61.2, 72.1)	63.4 (56.1, 69.6)	61.6 (53.7, 68.8)	59.6 (51.3, 65.2)	**<0.001**	-0.239	**<0.001**
Male sex, n (%)	116 (62%)	126 (68%)	133 (72%)	148 (80%)	**0.002**	0.107	**0.003**
BMI, kg/m^2	25.4 (22.6, 29.1)	25.7 (23.7, 29.3)	26.6 (24.2, 29.7)	26.7 (24.9, 29.1)	**0.009**	0.018	0.632
AF subtype, n (%)					0.15	-0.077	**0.049**
Paroxysmal AF	100 (54%)	107 (58%)	115 (62%)	120 (65%)			
Persistent AF	86 (46%)	78 (42%)	71 (38%)	65 (35%)			
**Medical history, n (%)**							
Hypertension	83 (45%)	70 (38%)	72 (39%)	69 (37%)	0.44	0.008	0.816
Diabetes mellitus	11 (6%)	15 (8%)	10 (5%)	8 (4%)	0.47	-0.019	0.565
Stroke, TIA or thromboembolism	21 (11%)	13 (7%)	9 (5%)	9 (5%)	**0.049**	-0.071	**0.037**
Congestive heart failure	24 (13%)	17 (9%)	18 (10%)	18 (10%)	0.63	-0.054	0.112
Ischemic heart disease	15 (8%)	14 (8%)	12 (6%)	9 (5%)	0.62	-0.011	0.759
Report of dyspnea	162 (87%)	147 (80%)	134 (72%)	135 (73%)	**0.001**	-0.111	**0.001**
CHA_2DS_2-VASc score					**<0.001**	-0.078	0.083
0	23 (12%)	47 (25%)	54 (29%)	76 (41%)			

1	51 (27%)	54 (29%)	60 (32%)	42 (23%)			
2	57 (31%)	45 (24%)	33 (18%)	45 (24%)			
3	27 (15%)	28 (15%)	29 (16%)	17 (9%)			
≥ 4	28 (15%)	11 (6%)	10 (5%)	5 (3%)			
**Echocardiography of right ventricle**							
TAPSE, cm (SD)	2.2 (0.5)	2.3 (0.6)	2.4 (0.5)	2.4 (0.5)	**<0.001**	0.173	**<0.001**
TR Vmax, m/s	2.4 (2.1, 2.6)	2.3 (2.1, 2.5)	2.3 (2.2, 2.5)	2.2 (2.1, 2.4)	**0.026**	-0.084	0.104
**Echocardiography of left ventricle**							
LVEF, %	57.3 (49.3, 62.4)	59.2 (52.0, 63.4)	58.6 (54.0, 63.3)	60.7 (52.4, 64.3)	**0.018**	0.085	**0.045**
GLS, %	15.8 (12.1, 18.5)	16.1 (13.2, 19.1)	16.8 (13.9, 19.3)	16.7 (13.6, 19.1)	0.13	0.075	0.096
LVEDV, mL	70.9 (59.3, 87.6)	75.3 (63.8, 91.5)	79.3 (63.3, 99.1)	84.9 (69.4, 103.7)	**<0.001**	0.027	0.541
LVESV, mL	30.1 (24.9, 41.9)	32.5 (25.6, 38.9)	32.4 (26.5, 42.4)	35.0 (26.9, 44.7)	0.11	-0.033	0.395
E/e'sr, cm	65.0 (53.2, 82.5)	64.8 (51.3, 78.7)	63.0 (48.0, 80.5)	63.6 (51.0, 82.6)	0.57	0.007	0.855
LV mass index, g/m^2	90.4 (74.0, 108.8)	89.5 (75.5, 103.6)	86.6 (75.6, 100.4)	93.2 (78.9, 107.4)	0.24	0.052	0.178
**Echocardiography of left atrium**							
LAD, cm	4.0 (3.7, 4.3)	4.0 (3.6, 4.4)	3.9 (3.6, 4.3)	4.0 (3.7, 4.4)	0.58	-0.009	0.802
LAV_{max}, mL	62.5 (48.4, 77.4)	61.0 (46.8, 74.5)	62.4 (45.4, 73.8)	61.2 (48.8, 76.5)	0.90	-0.024	0.505
LAV_{min}, mL	42.1 (28.7, 56.2)	35.8 (26.5, 53.6)	36.5 (26.1, 49.7)	37.2 (27.9, 47.4)	0.13	-0.046	0.225
$LAVi_{max}$, mL/m^2	31.1 (24.8, 39.3)	30.2 (23.3, 36.2)	28.5 (22.8, 35.7)	27.5 (23.0, 35.4)	**0.042**	-0.057	0.128
$LAVi_{min}$, mL/m^2	21.2 (15.0, 28.2)	18.2 (12.5, 25.3)	17.2 (12.1, 24.7)	16.7 (12.9, 22.5)	**0.004**	-0.076	0.052

PALS, %	17.2 (11.6, 25.0)	19.8 (12.3, 26.5)	22.0 (15.4, 27.8)	22.3 (15.5, 28.4)	**<0.001**	0.093	**0.036**
PACS#, %	10.3 (6.8, 14.8)	10.6 (8.1, 15.2)	12.0 (8.0, 14.7)	12.5 (8.4, 14.9)	0.28	0.083	**0.045**
LACS, %	11.0 (8.3, 14.0)	12.1 (9.5, 16.3)	12.2 (9.8, 15.9)	12.5 (9.1, 16.6)	**0.015**	0.024	0.534
**Computed tomography**							
Pulmonary artery diameter, mm	26.5 (24.0, 29.4)	26.4 (24.2, 29.1)	26.7 (24.6, 29.4)	26.9 (24.2, 29.3)	0.73	0.027	0.440
Ascending aorta diameter, mm	33.2 (30.9, 36.0)	33.6 (31.1, 36.3)	33.8 (31.2, 36.6)	33.7 (31.0, 35.7)	0.85	0.041	0.255
PA:A	0.8 (0.7, 0.9)	0.8 (0.7, 0.9)	0.8 (0.7, 0.9)	0.8 (0.7, 0.9)	0.47	-0.001	0.975
%LAA-950	0.14 (0.057, 0.46)	0.086 (0.025, 0.21)	0.039 (0.017, 0.11)	0.029 (0.010, 0.11)	**<0.001**	-0.245	**<0.001**

Data are expressed as median (IQR) unless otherwise specified.

* Adjusted for age, sex, heart rate, AF during echocardiography, and emphysema.

# Only includes patients in sinus rhythm during echocardiography (n=547).

Abbreviations:

AF = atrial fibrillation; BMI = body mass index; CHA_2DS_2-VASc = score for congestive heart failure, hypertension, age, diabetes, stroke, vascular disease, and sex; E/e'sr = ratio of early mitral inflow velocity to early diastolic strain rate; GLS = global longitudinal strain; LACS = left atrial strain during conduit phase; LAD = left atrial diameter; LAV = left atrial volume; LAVi = left atrial volume index; LV = left ventricular; LVEDV = left ventricular end-diastolic volume; LVEF = left ventricular ejection fraction; LVESV = left ventricular end-systolic volume; PACS = peak atrial contraction strain; PALS = peak atrial longitudinal strain; PA:A = ratio of pulmonary artery diameter to ascending aortic diameter; TAPSE = tricuspid annulus plane systolic excursion; TBV/TLV = total blood volume normalized to total lung volume; TIA = transient ischemic attack; TR Vmax = maximal tricuspid regurgitation velocity; %LAA-950 = percentage of emphysematous lung tissue.

Figure Legends

Supplementary Figure 1:

Title: Pulmonary vascular volume by AF subtype.

Caption: Box plot presenting the median (solid mid-line), 25th to the 75th percentile (box portion), and 2.5^{th} to the 97.5^{th} percentile (whisker portion) of TBV/TLV and BV10/TLV stratified by AF subtype. Linear regression analysis was utilized to assess p for trend values, while Wilcoxon rank-sum test was used for pairwise comparisons between groups.

Abbreviations: AF = atrial fibrillation; BV10/TLV = blood volume of pulmonary vessels <10 mm^2 in cross-sectional area normalized to total lung volume; IQR = interquartile range. PAF = paroxysmal atrial fibrillation; TBV/TLV = total blood volume normalized to total lung volume.

Supplementary Figure 1:

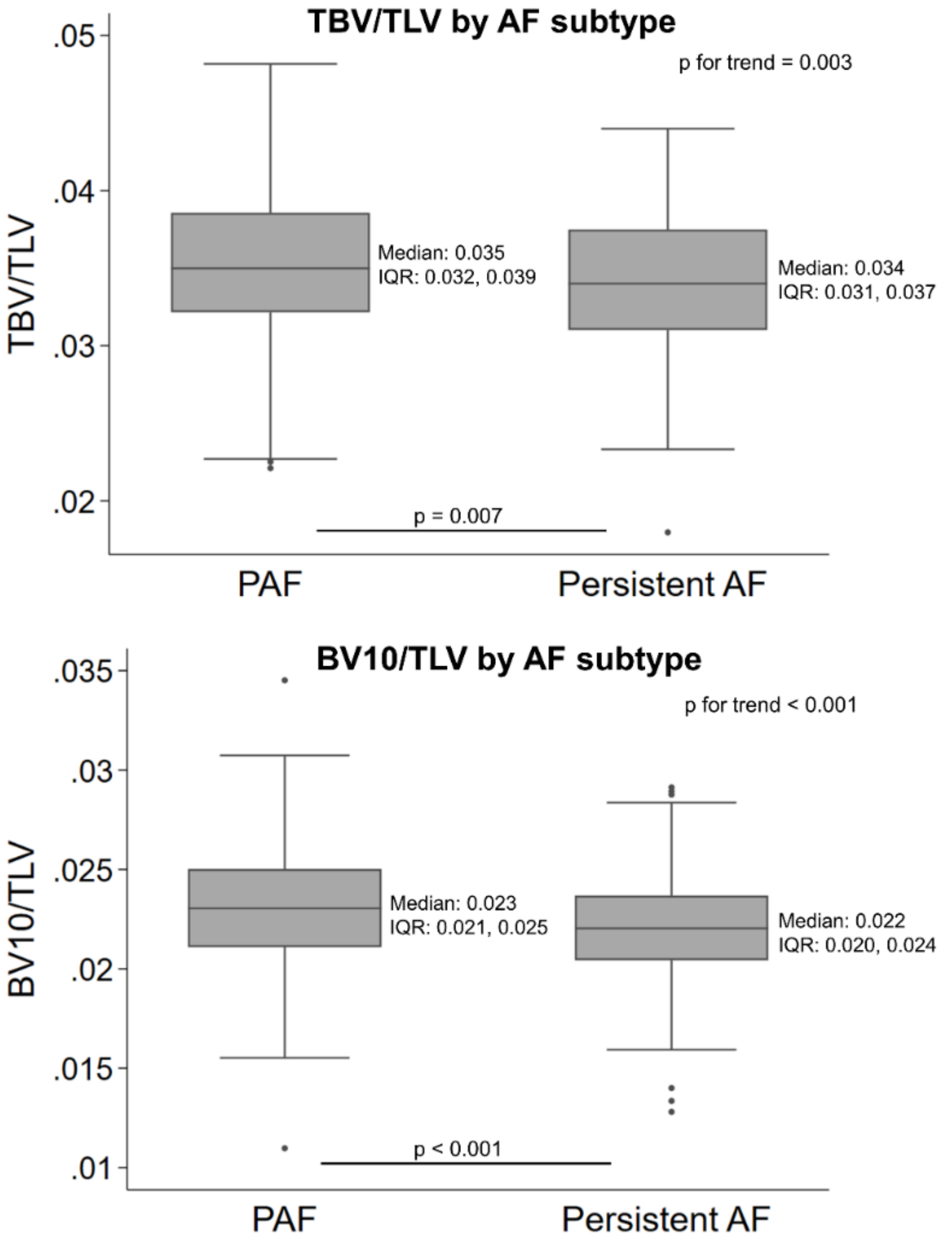

Supplement: Supplement [file NIHMS2182249-supplement-Supplement.pdf]
